# Hospital Practice

**Published:** 1828-04-01

**Authors:** 


					1828] ( 553 )
$etfdtope;
OK,
CIRCUMSPECTIVE REVIEW.
" Ore trahit quodcunque potest, atque addit acervo.'
[22d MARCH, 1828.]
HOSPITAL practice.
!. GLASGOW ROYAL INFIRMARY.
Spinal Disease.
Mr. Auchincloss, Surgeon to the Glasgow
Royal Infirmary, has published a case in
the first number of our Glasgow cotem-
porai-y, which he considers to be valuable,
as tending to confirm the opinion first
suggested by Mr. Brodie, regarding the
difference in the nature and treatment of
Ulceration confined to the intervertebral
Substance?and ulceration affecting the
cancellous structure of the bone. The
following case is offered in illustration.
Case. " 7th Sept. James Graham, by
trade a gardener, aged forty-five, of rather
a spare habit, though healthy, met with
an accident five weeks ago, the nature of
which was as follows :?While standing
on a bench, four feet high, with his arms
extended in the act of pulling fruit from
a tree, he happened to lose his balance,
when, to support himself, he caught hold
of one of the branches. After swinging
a few seconds, the branch broke, and he
alighted perpendicularly on the ground,
a height of about two and a half feet.
He felt little uneasiness at the time of
the fall, which occurred at three o'clock,
p. m. and, accordingly, continued at his
employment during the remainder of
working hours. Since the following
morning, he has complained of a sense
of tightness around the lower part of the
chest, with pain in the right hypochon-
drium, particularly aggravated by mo-
tion, though not in the least impeding
full inspiration. Pulse 76, weak ; bowels
moderate; other functions natural. Was
bled from the arm three different times
by a medical gentleman, whose advice
he requested at the time; and has had
two blisters applied to the region of the
liver, but without producing any good
effect;
" The impression on my mind, from a
very cursory examination of his ailments
on first seeing him, was, that the liver,
or muscles in its vicinity and about the
back, had sustained some degree of strain-
ing by the fall. He was again blistered
and otherwise treated as for simple in-
jury of those parts, during the first fort-
night. On one occasion, he experienced
much relief from cupping near the spine
on the right side, but was in no other
respect benefited by the treatment.
" The symptoms of his complaint had
become now much more apparent, and,
on a more careful inquiry being made,
were found to be connected with slight
curvature of the sixth, seventh, and eighth
dorsal vertebrae. The convexity of the
curve was turned outwards, and he seemed
to suffer but little uneasiness in the part
when firmly pressed upon. The follow-
ing symptoms were then noticed:?
Numbness of the lower extremities, with
much bodily weakness and inability to
sit, for even a few minutes, in the erect
posture; scanty secretion of urine, cos-
tiveness, and some swelling of the ab-
domen ; constriction and pain around the
lower part of the thorax, most acute on
the right side ; loss of appetite, thirst,
and a weak accelerated pulse.
" On the following day, the caustic
potash was applied on each side of the
protuberance, but without occasioning
relief at the time, or on the sloughs sepa-
rating, four days after. He became daily
weaker, and expired on the 21st of Oc-
tober, exactly eleven weeks and two days
from the date of his falling from the tree.
For some time previous to death, his belly
Vol. VIII. No. 16. 3 0
554 Periscope; or, Circumspective Review. [March 22
was tympanitic and swollen to a great
size ; and he was much troubled with
feelings of numbness in the left shoulder.
" Inspection.?The pleurae were ex-
tensively adherent on both sides. The
lungs, with the exception of the posterior
part of the right, which was collapsed,
exhibited their natural appearance and
structure. There was an abscess, con-
taining about five ounces of curdy-looking
purulent matter, in the posterior medias-
tinum. This was situated in front of the
sixth, seventh, and eighth dorsal ver-
tebra, and encroached considerably on
the right side of the chest. The body of
.the seventh vertebra was almost wholly
destroyed by ulceration; but this had pro-
ceeded much farther on the right than
the left side. The bodies of the other
two vertebrae were also partially absorbed.
In no part, however, had the disease ex-
tended to any of the intervertebral car-
tilages, all of which remained in an en-
tire state. Notwithstanding the extent
of destruction, the osseous structure both
of the diseased vertebra and of those in
the neighbourhood, was of its natural
hardness and colour. The liver was
healthy, as also the other abdominal
viscera."
There are very few cases of this kind
on record. Mr. Brodie has offered two,
in his treatise on diseases of the joints.
" In treating of this disease, with the
view of distinguishing it from a similar
affection originating in the intervertebral
substance, Mr. Brodie regrets that there
should exist no better criterion than the
following, to direct us in our judgment.
In ulceration confined to the cartilage,
he remarks, the patient is benefited al-
most immediately on the issues being
made, or, at least, feels himself uniformly
easier after each application of the caus-
tic ; but this does not take place in the
other species, for in it issues of every
sort constantly fail in affording even the
slightest relief. Moreover, he supposes
that the form of disease which begins in
the substance of the bone, is generally
rapid in its progress, being more imme-
diately followed by suppuration than that
which commences in the intervertebral
substance; and that, in consequence, des-
truction of the contiguous vertebra takes
place to a much greater extent in the
one species of disease than in the other.
* But farther than this,' says that excel-
lent surgeon, * nothing which I have
hitherto observed enables me to point
out any other circumstances, in which
the symptoms of these different diseases
differ.'
" It affords me much pleasure to be
able thus far to bear testimony to the
truth of Mr. Brodie's statements, respect-
ing the diagnosis of these two affections.
From a knowledge of the facts adduced
by him, and to which I have now referred,
I was enabled, in the present instance,
to form an opinion as to the true nature
of the disease, which was afterwards fully
justified by the dissection. The case cer-
tainly affords a very striking illustration
of the great rapidity of the disease, and
of the utter, inefficiency of caustic issues
as a means of cure."
Case of Compound Fracture.*
A great improvement has been effected
of late iii the treatment of compound
fractures in this. Infirmary by Dr. Young,
in consequence of the continuation of the
dressings, the method of treatment re-
commended by Baron Larrey.
John Durrough, set. 32, labourer, was
admitted into the Infirmary, July 16th,
1827, under the care of Dr. Young,
having received a few hours previously a
compound fracture of the left leg, and in-
ferior maxillary bone. Inferior extremity
of tibia is fractured transversely, about an
inch above ancle joint?fibula is fractured
obliquely immediately above malleolus?
ancle joint is laid open on its outside an-
terior and posterior to extensor tendons,
the ligaments on inside of joint are not
injured. There is a wound of the in-
teguments, extending from external mal-
leolus, obliquely upwards and backwards,
and continued round to inner malleolus,
communicating with a wound which had
been inflicted on the inside of ancle.
Along the outside of joint there are many
bony particles, which have been detached
from the extremities of tibia and fibula.
The ancle joint is but very slightly dis-
placed, and the large arteries and all the
tendons around the joint have escaped
and are covered by the fascia. Lower
jaw is fractured obliquely a little to right
* Mr. Plymsoll's Clinical Reports.
Glasgow Infirmary.
1828] Incisions in Erysipelas. 555
side of symphysis?there is. great ten-
dency to displacement, as the fractured
extremities separate from each other, ex-
cept when the mouth is keptopen. Under
chin there is a wound an inch long, which
communicates with fracture. A consul-
tation was immediately held on this case,
and from the extensive and complicated
nature of the injury and its proximity to
the joint, and the joint itself having been
involved, immediate amputation of the
limb was determined on. The patient,
however, would not undergo the operation.
Pledgets of oiled caddis were applied to
wounds, and retained by adhesive plaster,
and the leg was then put up in splints.
2Jtli, Splints and dressings have been
removed to day. For the first time wounds
have a healthy granulating appearance,
with a little healthy pus on them. No
redness, pain or tension of surrounding
integuments?splints and dressings con-
tinued. August 2nd, Splints and dres-
sings have been again removed?wounds
continue to improve?limb can be freely
moved about for the application of ban-
dages. 23rd, Has had rigors?erysipe-
latous redness 0:1 fore-part of ancle, and
absorbent vessels about knee joint are in-
flamed?leeches were applied with great
effect?and pain and fever have dimi-
nished. Scarcely any constitutional de-
rangement occurred subsequently. Sep-
tember 1 Oth, Wound at outer ancle
healing rapidly?that at inner ancle has
cicatrized?bones have re-united?straps
and bandage. October 6th, Dismissed
cured.
Case of Ununited Fracture cured by
Pressure.
Daniel M'Vey, ajt. '44, porter, was ad-
mitted September 21st, 1827, under the
care of Dr. Maclachlan. He had a few
hours previously, whilst in a state of in-
toxication, come in contact with the
leader of a stage-coach which was driving
on rapidly, by which he was thrown down,
and the coach passed over him, inflicting
an oblique fracture of the left humerus.
There was a good deal of swelling around
the fracture, and the broken ends of the
hone rode to a considerable extent. The
arm was put in splints, which were re-
moved at the usual time, but no union
had taken place. The splints were re-
applied, but still without elFect. Dr.
Machlachlan having retired from the
hospital, the case was consigned to the
charge of Dr. Anderson, who had recourse
to pressure. Lrnen compresses were laid
over the fractured ends of the bone, and
splints and bandages were afterwards ap-
plied. A decided improvement was suc-
cessively observed, in the course of the
subsequent examinations, and by the 1st
of January, 1828, the cure was complete.
2. BARTHOLOMEW'S HOSPITAL.
Erysipelas treated by Incisions.
Two cases, treated in this way, have
lately been reported in the Medical and
Physical Journal, from Bartholomew's
Hospital, one of which is said to have
been lost by subsequent haemorrhage, in
consequence of the neglect of the nurse.
The first case was that of a man, aged 70
years, who came under the care of Mr.
Lawrence, for phlegmonous erysipelas of
the right leg and thigh, extending to the
groin. Three incisions were made?one
in the thigh, two inches long?another,
three inches long in the calf of the leg?
and a third, smaller than either, just below
the last. Much purulent matter escaped,
and large sloughs were discharged from
the wounds, without much hajmorrhage,
and with decided relief. In the night,
however, the man appeared to be dying;
but was recruited by ammonia, brandy,
and other stimulants. He recovered.
In this case, it appears that Mr. Law-
rence had recourse to incisions " by in-
stalments," which he so much ridiculed
in the practice of others.
Case 2. An old man came into Bar-
tholomew's in the beginning of January,
with erysipelatous inflammation of the
leg and foot, succeeding a blow. The
usual treatment not appearing to produce
benefit, Mr. L. made an incision, about two
inches long, " across the back of the
foot," and as there was but little bleeding
at the time, it was encouraged by fomen-
tations and a poultice, it appears that
haemorrhage soon afterwards came on,
and the nurse took no notice of it till
nearly a quart of blood was lost! The
house-surgeon was then summoned, and
had great difficulty in stopping the bleed-
ing. From this time, the patient, though
rallying a little occasionally, gradually
3 0 2
556 Periscope; or, Circumspective Review. [Marcfi 2$
fell back, and died nine or ten days af-
terwards.
We would not say positively, that this
man died in consequence of the haemor-
rhage, seeing that the patient was old?
that the disease in such subjects, is gene-
rally hazardous?and that the man lived
nine or ten days after the haemorrhage.
The neglect, of course, falls on the nurse?
but we cannot help wondering that the
incision should have been made " across
the back of the foot," as surgeons gene-
rally prefer to make wounds in the direc-
tion of vessels, nerves, and tendons,
rather than across them. Perhaps the
report is inaccurate, or there was some
specific reason for the mode of incision,
which is not stated. The case shews
that, after incisions in erysipelatous parts,
the most rigid instructions should be
given to the house-surgeon, and the case
never left to the sole charge of a nurse.
Axillary Aneurism.
In No. 286 of the Lancet, there is but
one report from all the London hospitals,
but like the single fault of poor Hickey,
the attorney in Goldsmith, " that one is
a thumper." It occupies no less than
seven dull columns of close print, and
the unfortunate ? readers may well ex-
claim with the frogs in iEsop, " ah !
Mr. Reporter, this may be sport for you,
but it is death to us!" As we are not
paid by the yard, we shall make some-
what shorter work of it than the Lancet,
and indeed we notice the thing more for
the sake of what fell from Mr. Lawrence,
than for any great interest in the case
itself.
J. L. set. 39, a very muscular man,
received a blow on the shoulder, which
was followed by pain, and numbness in
the arm. In the course of three or four
months the limb began to swell, and
about a month or so, prior to his admis-
sion, he noticed a swelling and beating
about the right shoulder, attended with
excessive pain. He was first treated for
rheumatism, and subsequently bled and
leeched.
He entered Bartholomew's on the /th
Sept. under the care of Mr. Lawrence,
with an imperfectly circumscribed elastic
swelling, " possessing all the characters
of an aneurism," its upper edge being felt
about two inches above the internal end o!
the clavicle, and " its lower boundary,
where the three upper ribs are closely
involved, being nearly indefinite." There
was no discolouration of the skin, but
the veins were distended; pulsation very
powerful in the upper part of the tumour,
not perceptible in the lower; limb swol-
len ; little or no pulse at the wrist of that
side ; much pain on the inner side of the
arm, and numbness of the fingers ; voice,
respiration, &c. natural ; pulse of the
radial artery 70, firm, and full. He was
ordered to be kept quiet, purged, and
digitalis was administered. He was bled,
also at intervals, and went on, sometimes
better and sometimes worse, until the
latter end of November, when the pul-
sation in the tumour became less distinct,
and soon altogether ceased?the tumour
became stationary and firmer?pulse at
the radial artery, which had increased,
was less distinct?and the pain and swel-
ling of the limb were so far diminished
that he could move it more freely, and
grasp more firmly. Previous to this, the
stethoscope had been applied, but no
disease of the heart, or its large vessels
within the thorax could be detected. The
amendment went on " progressing" until
the month of January, 1828, when he
began to be affected with slight cough1
and irritation about the trachea, and on
the 15th, the tumour suddenly and rapidly
increased; the arm swelled, and the cough
was very troublesome. In spite of bleed-
ing and the application of cold, the dis-
ease increased; the tumour acquired ar*
immense size ; a slough formed over the
scapula; he was forced to swallowdrachm
doses of laudanum to procure sleep, and
on the 9th of February, death put an end
to the poor fellow's tortures.
Sectio Cadaveris. This is detailed,
like the case, with great prolixity; suffice
it to say that the aneurism was of enor-
mous size, reaching from the sternum to
the inferior angle of the scapula, and
containing three pints of recently coagu-
lated blood, with a little fibrine adhering
to its sides. These were made up at the
upper portion, of the proper arterial coats
with little adherent coagulum, and below,
of the surrounding textures, muscles,
bone, &c. lined with a coagulum, not
exceeding half an inch in depth at the
thickest. The upper rib was rough and
partially absorbed, the second denuded,
1S28] . External Iliac Tied. 557
and the aneurysmal sac extended between
the two into the chest, distending the in-
tercostal muscles, thickening the pleura,
and, at its convexity, adhering to the
lung. The sac originated from the axil-
lary artery, an inch below the origin of
the subclavial branches, an interval of
five inches remaining between the upper
and lower orifices of the artery. The
latter was about one inch above the giving
off of the sub-scapular. The axillai-y
plexus of nerves was involved in the
disease, and some of the nervous chords
flattened like tapes, but there was no
disease of the heart or great vessels.
Mr. Lawrence considered that the ex-
tension of the tumour above the clavicle,
precluded the ligature of the subclavian
beyond the scalenus ariticus muscle. The
only chance^ according to Mr, L. would
have been the operation on the innomi-
nata, but " there is little encouragement
to perform this operation, and the chance
of saving life, is probably no greater than
what is derived from the hope of a spon-
taneous cure." "The artery might have
been tied beyond the tumour. The liga-
ture, in that case, must have been placed
beyond the origin of the infra-scapular,
and circumflex arteries. Mr. Lawrence
observed, that we have hitherto no direct
facts in favour of such a proceeding ; and
that, in his opinion, the probabilities ivere
strongly against the attempt
In this sentiment of Mr. Lawrence we
entirely agree. We have stated, again
and again, that almost the only artery to
which Mr. Wardrop's operation (as it is
most absurdly termed) is applicable, is i
the carotid, because that vessel gives off ]
no branches betwixt the ligature and the i
aneurismal sac. We are glad to find that <
Mr. Lawrence makes a proper estimate <
of the operation, and is not one of those 1
good easy folks, who imagine that its r
promulgation is to constitute a kind of 1
Hegira in surgery, and are eternally <
chaunting their cWa/i-iV/a-aWa&'s to the s
glory, not of the Prophet, but of Mr. ^
Wardrop ! 1
P. S.?In this report, Mr. Lawrence is i
made to say that, no patient has hitherto <
recovered after ligature of the innomi- i
nata. What will Dr. Valentine Mott say
to this ? If his statement be correct,
there was a recovery from the operation,
as far as the operation itself was con- ]
cerned. The man died many weeks after
the ligature had been applied?and after
walking about the grounds of the hos-
pital.
3. NOTTINGHAM GENERAL
HOSPITAL.
Femoral and Popliteal Aneurism?
External Iliac Tied.*
Case 1. W. S, set. 42, after having taken
a long walk, when in a bad state of health,
discovered a pulsating tumour in the
thigh, and soon afterwards another in the
ham of the same side. Both gradually
increased until about the middle of July,
nearly eleven months after their first ap-
pearance, when that in the thigh became
very painful, and enlarged rapidly, whilst
the tumour in the ham slowly diminished,
and from this time no pulsation could be
felt in either. On admission into the
hospital, August 24, 1824, the upper
aneurism was found to extend from two
inches beneath Poupart's ligament to
nearly half way down the thigh ; it was
hard, marked with dark purple spots, and
no pulsation could be discovered in it,
but on applying the stethoscope, a sound
like water forced through a narrow tube
could be heard synchronous with the pulse
at the wrist. The pulse in the artery
above was weak, and none could be dis-
tinguished in the popliteal aneurism. He
was kept quiet in bed, and cold applied,
under which treatment both tumours ma-
terially diminished in size, and in great
measure subsided. On the 17th Septem-
ber, however, after making some exertion,
all the former symptoms returned with in-
creased violence, and it was determined in
consultation to tie the external iliac, which
was done on the 22d, by Mr. W. Wright.
The steps of the operation we need not stop
to describe, but we may mention that on
exposing the artery, two large veins were
seen running obliquely over it, one of
which was ruptured in passing the needle
beneath the vessel. On the 25th, The
wound was dressed, and found to have
united above and below, but to remain
open in the centre. The tumours dimi-
nished in size, and became softer, but in
* Mr. Booth Eddison's Report, Med.
Repository for March, 18^8.
558 Periscope ; or, Circumspective Review. [March 22
the commencement of October, he began
to be affected with cough, and occasional
palpitation at the heart. The ligature
had not separated on the 10th January,
(four months and a half after its appli-
cation) and on that day it broke short in
the attempt to get it away. On the 18/A
the wound had healed, and he was made
an out-patient, but on 12th April, his
health was bad, there was a little descent
of intestine through the opening in the
external oblique, and he was troubled
with so much dyspnoea, palpitation and
cough, that he was re-admitted into
hospital, when he died suddenly on the
morning of the 15th.
Sectio Cadaveris. The tumours in
the thigh and ham were diminished in
size, firm, elastic, and the former co-
vered with a compact fascia. The ex-
ternal iliac was pervious from its origin
to the ligature, when it was completely
obliterated, and the knot and loop of the
ligature, which it .will be remembered
had been broken short, were found by
the side of the vessel, lying in a cyst,
" resembling an inguinal gland." Below
the ligature, the artery was pervious to
its entrance into the tumour, which con-
tained dark coloured, fluid stuff, and its
parietes were livid with layers of coagu-
lum of the same colour. The tumour
was almost filled up at its posterior
part, and about the entrance and exit
of the artery it was firm and cartil-
aginous. Between this and the aneu-
rism in the ham, which was of the
size of a pullet's egg, and contained
much the same sort of semi-fluid matter,
the artery was thinner in its coats, but
presented no other pai'ticular appearance.
The cause of death was found to have
been the rupture into the left side of the
thorax, of a large aneurism of the arch of
the aorta.
It is worthy of remark, how very long
the ligature was in separation, or rather
that it did not separate at all. The upper
tumour, we are told, commenced about
twoinches belowPoupart'sligament, con-
sequently, it was in all probability confined
to the superficial femoral artery. Under
these circumstances, it would be satis-
factory to know what prevented the ope-
rator's tying the common femoral, instead
of the external iliac. Surely the former
operation is much the more simple of the
two, and likely, we should imagine, to be
quite as effectual, for the coats of the
vessel do not appear to have been more
affected in the femoral than in the iliac.
The operation seems not to have done
very much towards the cure of the aneu-
rism, for the artery was pervious from
the ligature to the sac, through which,
if we understand the report aright, there
was still a passage open for the blood.
The diminution of the tumours upon the
patient's first admission into hospital,
evidently shewed an attempt at a spon-
taneous cure, and it is not improbable,
that had the local application of cold
been persevered in, and the Valsalvan
method put in force, the subsidence of
the femoral aneurism would have taken
place to nearly as great an extent as it
did after the ligature of the iliac artery ;
at any rate, such would be a fair case for
the trial of rigid depletion. There is a
considerable advantage attending this
plan, viz. that it would be adapted alike
for the external and internal aneurisms,
and thus be killing two birds with
one stone, whereas the ligature on the
external iliac, would certainly tend to
aggravate the aneurism of the aorta. In
the pi'esent case, the symptoms of the
latter disease only made their appearance
after the performance of the operation
for the former.
Case 2.?Paralysis of the left Side of
the Body, from Fungus on the Dura Mater.
J. H. set. 28, received a blow with a stick
on the left side of the head. In the course
of two or three days he suffered conside-
rable pain in the part, and an abscess
formed there, exposing dead bone. On
the 1st of April, two months after the
infliction of the injury, he was made an
in-patient, under the care of Mr. Atten-
burrow. At this time, he was labouring
under paralysis of the left arm and leg,
and had had several fits, in which he was
much convulsed. Some portions of the
outer table of bone had been taken away,
and now the whole diseased mass of the
parietal bone (a very considerable portion)
was removed by the trephine and Hey's
saw. This operation disclosed a fungus,
sprouting from the surface of the dura
mater, from which membrane it was dis-
sected off by the scalpel, the wound dressed
with lint and oiled silk, and the parts kept
cool by spirit lotion.
On the night of the 2nd, he had two
1828] Thoracic and Abdominal Aneurism.
559
fits, and, on the 3d, lie was in a kind of
comatose state, the left arm paralyzed,
the pupils dilated, and the expression wild.
A blister was applied to the nucha, and
he took sulphate of magnesia, and vinum
ipeeacuanhaj with relief to the above symp-
toms. On the 5th he was rather inco-
herent, and was bled to ?xiv. which was
repeated on the 7th; and, on the 9th, the
power over the left side was gradually re-
turning. The wound went on granulating
tolerably kindly, and, on the whole, there
was perceptible amendment, when, on
the 18th, an erysipelatous swelling ap-
peared upon the face. This was treated
by a small blood-letting, with purgatives
find antimonials, and, on the 22nd, the
erysipelas had nearly disappeared. The
fits, however, now returned, small por-
tions of bone became exposed, twitching
and tingling of the left arm became trou-
blesome, and he began to be attacked
with severe cough, pain in the left side,
and expectoration. This carried him off
on the eighth of June, the wound, at that
time, not having healed, but being healthy,
and the head-symptoms having disap-
peared for some time previously. No ex-
amination was allowed.
This case may be appended to two,
somewhat similar, detailed in our last
fasciculus, as well as to others, which we
have quoted from the writings of Aber-
crombie and O'Halloran.
A case is detailed by Mr. Eddison, in
which the tongue was removed, in con-
sequence of scirrhus. Mr. Wright per-
formed the operation, by drawing out the
tongue with a hook, (the jaws being se-
parated with a wedge) then making an
incision, from the back of the induration
to the tip of the tongue, and with a pair
of curved scissors dividing it beyond the
tumour, so that nearly the whole of the
scirrhus was removed; what did remain
was pared off by the scalpel. The wound
healed ; but symptoms of a return of the
disease have unfortunately shewn them-
selves.
4. HOSPICE GENERAL.
Aortic Aneurisms.
Case. M. D. a soldier, aged 40 years,
was discharged from the service in 1814,
having gone through immense fatigues in
his previous campaigns. He retired to
his native place, apparently in good health,
with the exception of some palpitations,
which he experienced after using any
strong exercise, or getting into a passion.
These he had felt ever since the age of
25 years. In 1824, a new train of symp-
toms made their appearance:?these were
obstinate constipation, accompanied by
pain in the left hypochondrium. The
defaecation, which took place only once
a week, or once a fortnight, was attended
with violent straining. After this con-
stipation had lasted about two months;
the pain in the hypochondrium became
much aggravated, attended with tume-
faction of the abdomen, colicky painsi
anxiety, shrinking of the features, vomit-
ing, great discharges of air from the
bowels and stomach, followed by tempo-
rary relief. The physician in attendance
now observed a tumour in the left side of
the abdomen, and, as the patient was in
a state of extreme emaciation, the medical
attendant, and some others who were con-
sulted, concluded that there was a scirrhus
of the colon. The patient getting worse
and worse, the man entered the Hospice
General. A few days previously, it was
accidentally perceived that the left side
of the chest was bulged out, and presented
considerable pulsation?in short, there
was evidently an aneurismal tumour, the
existence of which had never before been
suspected. Three days after entering the
hospital, (June, 1825) the man suddenly
expired while lying in his bed, reading a
book. I
Disscction. There was very little effu-
sion into the abdomen. The liver, sto-
mach, and intestines, were sound. In
the left side, the spleen, kidney, and colon
were displaced, and thrust forward by a
considerable tumour. The left thoracic
cavity contained a pint of sanguinolent
fluid, and a clot of blood, weighing fouf
pounds. The lung was sound, but pushed
upwards and to the right side. In this
side of the chest was seen the summit of
the tumour, which was detected pre-
viously in the abdomen, with a small
opening into the thorax, around which
was some coagulated blood. The extra*
vasation into the thorax had evidently
come through this opening. In the right
side of the chest, the lung was sound>
and a small tumour was seen projecting
from the descending thoracic aorta. The
heart was small, and unaltered. The an-
terior parietes of the aorta were sound;
560 Periscope ; or, Circumspective Review. [March 22
but, opposite the tenth dorsal vertebra,
there was an opening, an inch and a half
in diameter, which led into the little tu-
mour above described in the right side,
and also into the great tumour which was
seen in the side of the chest and abdomen.
Both were, of course, false aneurisms, the
larger being, in some places, three or four
inches in diameter. Part of it was above,
but the greater part below, the diaphragm.
The parietes of the large tumour were
principally formed of condensed cellular
membrane?in some places, of ribs and
spine?the interior presenting various
layers of fibrine, suoh as are generally seen
in false aneurisms. Several of the verte-
brae were carious, from the pressure of
the aneurism. It appears, though the
description is not very clear, that the tu-
mour in the right side was the original
aneurism?that the parietes of this tu-
mour gave way, and the larger aneurismal
pouch was then formed. Finally, this
last gave way, and death was the imme-
diate consequence.?Rev. Med,
5. MILITARY HOSPITAL.
Curious Case of Hepatic Disease.
The following case, with the dissection,
is recorded in Mr. Annesley's great work,
recently published. It forms the subject
of the 14th plate in that magnificent pub-
lication.
Case. Ed. Gorman, serjeant-major,
aged 45 years, an old soldier, a hard
drinker, and many years resident in India,
had suffered repeated attacks of chronic
hepatitis, and had been several months in
ill health previous to his admission into
hospital, on the 5th June. His legs were
then much swelled?tongue foul-?bowels
constipated?complains of a confused sen-
sation of pain about the loins?general
torpor of upper and lower extremities.
He was ordered aperient pills at night,
containing a proportion of calomel. 6th.
Tongue foul-r-stools scanty, of an olive-
green colour. Purgatives. 7 th. Stools
more natural?other symptoms the same
?purgation continued. 8th. Much bet-r
ter. The swellings of legs subsided?
pulse good, tongue clean?numbness in
the hands continues, but is abated in the
legs. Purgation. 9th. Stools more fse-
culent?functions of limbs restored?com-
plains of " a painful heat,in his bowels.'*
Purgation. 10/Zs. Stools more copious,
faeculent, of a darker colour. Purgation.
He went on, sometimes better, sometimes
worse, till the 2'2d of June, when death
closed the scene; The numbness and
pain in the extremities continued more
or less till the last.
Disscction. A considerable quantity of
lymph was effused over the brain, which
was soft and pale. Three ounces of fluid
in the ventricles. " On opening the ab-
domen and thorax, the whole abdominal
viscera were found inflated." The liver
was very pale, and extremely small. On
its surface was a large fissure, that marked
the situation of a former abscess. The
structure about this part was changed
into a kind of cartilage. " The internal
constitution of the liver was otherwise
natural. When divided in the situation
of the cicatrix, the cicatrization of the
substance of the organ was evident through
the greater part of its thickness." Some
constrictions were observed in the small
intestines, which were otherwise healthy.
There was no ulceration in the large in-
testines, but the colon took several most;
extraordinary turns and contortions, from
one side to the other, before it ended in
the rectum. It appears from the plate?<
and, indeed, it must have been, greatly
elongated.
Remarks. As the cicatrix in the liver,
in this case, was not adherent to any part,
the abscess must have burst into the cavity
of the abdomen, and it would have been
very interesting to have learnt the partii
culars of that illness. There is another
case in the same work, where the cicatrix is
immense, but not quite free from adhesion.
This case we shall detail in a succeeding
periscope. Was not the torpor of the
limbs, in the above case, the consequence
of the efFusion going on within the head?
The man, in fact, died of the cerebral af-
fection, though that affection was pro-
bably induced by the hepatic disease?or
rather the mal-function of the liver.
MISCELLANIES.
I, Royal College of Physicians.
We have learnt, with considerable sur-.
prise, that meetings of the Fellows of
1828] Royal College of Physicians. '561
the Royal College of Physicians have been
held, for the purpose of deliberating on
measures to be taken against the Editor
of this Journal, on account of comments
lately published on the constitution and
laws of that body. The Censors of the
Royal College, in particular, have consi-
dered themselves as accused of dereliction
of duty, and of venality in the discharge
of their functions, by the observations
made in the article on medical education,
in the 4th fasciculus of this Journal. The
writer of that article was particularly cau-
tious in avoiding every expression that
might, in the smallest degree, bear upon
the personal conduct, or character of any
individual, or class of individuals, belong-
ing to the College. He confined his ob-
servations entirely to the effects of the
constitution and laws of the College, (as
handed down by their forefathers) on the
progress of medical science?and did not
even hint that these laws were either
made, or badly administered, by the pre-
sent race of Fellows or Censors. We un-
derstand that the Censors have considered
our observations as reflecting on them, in
their function of -examining physicians
for the licence, and as imputing to them
certain mercenary motives, in passing over
the said examinations, without proper at-
tention to the qualifications of the candi-
dates. No such interpretation can be
fairly put upon our remarks. We argued,
indeed, as an abstract principle, that the
receipt of a revenue from the disposal of
licences, in any corporate body, was a bad
principle?" a principle which must in-
cessantly prompt to extend the amount of
sale, without any (we ought to have said
adequate) reference to the qualifications
of the purchasers." This is the head and
front of our offending. We never said
that any Censor, of this or that year, was
seduced from his duty by the temptation
which the laws of his college threw in his
way. No. We carefully abstained from
any such insinuation. AVe insisted on the
evil tendency of the law; but never ac-
cused any set of men of giving way to
this tendency. We have no hesitation,
indeed, in saying, that the very Censor
who has moved the College to proceed
against us, has shewn that he could act
in direct opposition to this evil tendency
of the principle in question. So far from
giving, way to self-interest?or, what is
the same, the interest of his College?he
rejected one of the most eminent physi-
cians of this metropolis, and thus deprived
his College, for a time, at least, of the
emolument derivable from the licence.
The still more recent rejection of Dr.
John Mason Good, was another example
how far individuals will resist the influ-
ence of imperfect laws. But are we not
to censure a bad principle, because there
are men virtuous enough to act on an
opposite principle? Are the laws of the
College more sacred than the laws of our
country? Does anyone find fault with
Mr. Brougham, for condemning laws en-
acted by that very senate of which he is
a member ? If no man be allowed to
canvass or criticise a law that has once
been passed?then no law can ever be
abrogated afterwards. The College of
Physicians have often altered their bye-
laws?and surely this must have been on
a conviction that they were not good.
Why, then, should the College wish to
destroy the liberty of the press, by per-
secuting a licentiate who investigates the
tendency of other laws, not yet altered or
repealed ? We have never advocated the
cause of involution?we have never joined
in the cry?" down with the charter," of
this or of any college. No. We wish to
see the original charter preserved in all
its purity and simplicity?and we would
certainly wish to see the bye-laws, insti-
tuted from time to time, in accordance
with the spirit of the age in which they
are framed. It was sagaciously remarked,
by no less a personage than Sancho
Panza, that?" it is safer for one man
to steal a horse, than for another man to
look over a hedge." So we have found
it. Any word that drops from us, is tak-
en up and canvassed with the most rigid
scrutiny. But other writers may say what
they please, with impunity. If we had
put on our pages such a passage as the
following?and that, too, from the pen of
a most ardent advocate of the College,*
the beadle would have been at our door
in less than 24 hours !
" From the moment that the Corpo-
ration of Physicians submitted to this in-
fluence, (a recent influence which we
shall not name) they lost the high ground
on which their advocates had endeavoured
to place them ;?they ceased to be the in-
* Report says Mr. Ponblanc.
562 Periscope ; or, Circumspective Review. [March 22
flexible guardians of the principles trans-
mitted to them by their predecessors. They
had not adopted the reasonable rule, of
relaxing the rigour of their bye-laws, in
favour of superior talent ; but they sa-
crificed their independence to a petty in-
trigue
We ask the amiable and talented Pre-
sident of the College?we ask the Cen-
sors?we ask the Fellows, whether we
have ever said any thing so severe as
this ??The Editor of this Journal has
only to observe, in conclusion, that if the
President of the College will place twelve
unbiassed and impartial physicians in the
jury box, he will plead to the indictments
which are about to be brought against
him. But if he is cited before a tribunal,
each individual of which unites in his
own person the triple character of pro-
secutor, judge, and juror, he will be as
mute as the marble busts of Harvey, Sy-
denham, and other of his departed bre-
thren, ranged round the room where this
scene is to take place. If the Editor has
published, or suffered to be published,
false facts or illegitimate conclusions, the
press and the laws of the country are open
to the College for redress. But, if the
conditions of his licence preclude him
from all freedom of thought and expres-
sion that may not be pleasing to the
rulers of the said College, he will tender
his license to the President, and fall back
(if it be really a retrogradation) to that
station in medical society, in which the
greater and the best part of his life has
been spent. To him, it matters not whe-
ther Dr. or Mr. be prefixed to his name.
It is not the letter M, or the letter D?
or the combination of these two letters,
M.D., that can materially affect the re-
sidue of a life passed in the ardent (how-
ever unsuccessful) pursuit of medical
knowledge. If the College, therefore,
think that they shall enhance their au-
thority or dignity by erasing his name
from their list, he begs that they will not
permit any private or personal feeling to
interfere, for one moment, with the policy
of the measure.
2. Du. Kellie on Tubercles, and their
Effects on different Structures.
(Dr. Monro's Work on the Brain, vol. I.)
Dr. Kellie observes, that common tuber-
cles may be formed?attain a considerable
size?and long exist in any of the various
structures of the body, without producing
much trouble or sensible disturbance of
function. But, when they become ex-
cited, inflamed, or suppurated, then they
begin their work of disorganization in the
contiguous parts, and produce trains of
symptoms?more characteristic, however,
of the injured function of the matrix of
the tubercles, than indicative of the ex-
istence of the parasitic formations them-
selves.
" I have found tubercles in the serous
membranes, and imbedded in the sub-
stance of the liver, of the spleen, of the
kidney, of the uterus, of the lungs, of the
brain, and of the cerebellum, in subjects
in which no symptoms had indicated their
existence during life. In young people,
in children especially, from five to ten or
twelve years of age, it is not uncommon
to find tubercles of various sizes, and in
various states of progression, co-existing
in all or most of the organs in the same
subject, who had enjoyed perhaps good,
or very tolerable health, till within a very
few days of his death. I found them in
the brain, in the lungs, and in the perito-
neum of one boy of seven years of age ;
in the brain, in the medulla oblongata, in
the liver, in the spleen, and in the lungs
of another boy at the age of nine ; anil
yet these boys were active and well, and
continued at school till within fourteen
days of their death. Both died of Hy-
drocephalus acutus, passing, in the course
of fourteen days, through all the stages
of that disease, with nosographic regula-
rity. In the latter boy, there occurred,
indeed, during the last two days of his
existence, a paralysis of the right side,
and a spastic rigidity of the left arm, which
enabled me to conjecture the probable
existence, not of tubercles, but of inflam-
mation and softening of the substance of
the brain, as well as of the distension of
the ventricles by serous effusion. Accor-
dingly, besides the dropsy of the ventricles,
I found two tubercles, each of the size of
a garden-pea, hanging pendulous from
the tentorium, two others of the same
size in the medullary substance of the
* The Jurist, No. III. Jan. 1828. In
quoting this passage, we are far from agree-
ing with the writer of it, in his general
principles. But we adduce the passage,
to shew that even the College advocates
can see defects in College administration.
1828] Dr. Kellie on Tubercles. 563
light hemisphere, and one oblong tuber-
cle, of the size of an almond, suppurated
in its centre, and imbedded in the pons
varolii."
This fine boy, till attacked with head-
ache, fever, &c. about a fortnight before
his death, was apparently free from dis-
ease?was active, diligent, and clever at
his school! The following case is inte-
resting.
" On the morning of Thursday the
2d of August, about half-past eight o'clock,
I was requested to visit, as speedily as
possible, a boy of the name of Bell, re-
siding in Poplar Lane, who had a little
before been suddenly attacked with violent
convulsions. When I arrived, the con-
vulsive struggle was over, but there was
still an occasional subsultus of the mus-
cles of the face and of the arms. He was
insensible and comatose ; the pulse was
very rapid, and the heart was striking
against the side with remarkable vio-
lence. My inquiries were answered to
the following effect: That his age was
six years ; that he had been upon the
whole healthy, never had a convulsive fit
bef6re, was not liable to headaches, though
he was to coughs ever since he had had
measles ; that he was in perfect health,
in so far as could be discovered on Tues-
day ; that he had taken his food that day,
and had amused and occupied himself as
usual, going to bed at the usual time
without complaint; that, on Wednesday
morning, when dressing, he complained
for the first time of pain of the loins, and
of that only; that, continuing to do so,
and seeming listless and unwell, his aunt
had, towards noon, given him a dose of
Epsom salts, which operated four or five
times, but without relieving him; that, on
the contrary, though he kept out of bed
all day, he complained more and more of
the pain in the back and loins till night;
that, towards eleven, he fell asleep, but
frequently awoke and complained of this
pain, and at length, towards three this
morning, he seemed to suffer so much,
that Mrs. Nichol, a midwife, residing in
the same quarter, and an intimate of the
family, was called to assist him; that she
administered an enema, and observing
what she called a great working about
the breast, she applied two leeches to the
sternum ; . that the excruciating pain of
the back continued unabated ; that he
complained incessantly, and was ex-
tremely restless till eight o'clock this
morning, when the convulsive attack su-
pervened.
" Six ounces of blood were instantly
taken from the arm ; another enema was
then administered, and a purgative pow-
der was directed to be given in a spoonful
of gruel, so soon as the patient recovered
the power of swallowing.
" But he never recovered his sensibility.
The spasmodic twitchings of the muscles
of the face and arms continued to recur
at short intervals ; and at half-past noon
.there came on another general convulsion,
which continued, with little abatement,
till one o'clock, when he died.
" Next morning the body was examined.
The general appearance of the corpse was
that of a well-grown boy of his age. The
medulla spinalis, to which my attention
was first directed, on account of the ex-
cruciating pain complained of in the back
and loins, very carefully examined in its
whole length, exhibited no trace of disease
in its substance, its membranes, or its
vascular system. The spinal nerves were
equally sound.
" In the cerebrum I found three tuber-
cles, and in the cerebellum one. Of those
discovered in the brain, one the size of a
small chesnut was found adherent to the
arachnoid of the dura mater, and buried
in the substance of the convexity of the
posterior lobe of the left hemisphere, from
which it was withdrawn along with the
dura mater, on raising that membrane in
the usual way from the cerebrum.
" When the softened cerebral substance
torn from the brain by the removal of this
tubercle was washed from it, the tubercle
itself, of the size and shape nearly of a
chesnut, appeared somewhat nodulated
on its surface : it was very firm, of a
greyish colour, and invested by a mem-
branous cyst, having evident vascular and
membranous connections with the arach-
noid, and with the brain. Other two tu-
bercles were found, one in each hemis-
phere of the brain, imbedded in the me-
dullary walls of the lateral ventricles in
corresponding situations, a little above
and between the corpus striatum and tha-
lamus of either side."
Dr. Kellie has not met with any other
case, in which a fatal crisis has so sud-
denly occurred at so early an age. In
young people, the more common termi-
nation of such cases is by consequent.
564 Periscope; or, Circumspective Review. [March 22
arachnitis and hydrocephalus?in adults,
?by inflammation and softening of the ce-
rebral substance.
3. Popliteal Aneurism?Secondary
Hemorrhage.*
What a happy thing it is for the critical
tribe, that the surgical catalogue of ills,
contains two such diseases as hernia and
aneurism ! They are positively a Jour-
nalist's stock in trade, and were they,
by some dire mischance, but once obli-
terated from the nosologic chart, he
might lay aside his gall and grey-goose
quill for ever, and his occupation, like
?Othello's, would be gone! We have
-lately touched rather fully on the subject of
aneurism, but it is so wide and debateable
a ground, the questions, in many instances,
so obscure, and the disputes upon them
so hot, that?
To count them all, requires a thousand tongues,
A throat of brass, and adamantine lungs.
The subject of the present case, a
coachman, ait. 40, was admitted into the
Middlesex Hospital, on the 22d of Octo-
ber, under the care of Mr. Bell, labouring
under popliteal aneurism. He had always
enjoyed good health until three months
previously, when he had an attack of gout,
and about a month prior to admission,
had first noticed pain and stiffness in the
knee, which gradually increased. He was
kept quiet in the hospital, until the 3d.
November, when the swelling and tension
of the limb having subsided, the operation
of tying the femoral artery was had re-
course to. The vessel was secured at the
upper portion of the middle third of the
thigh, without much difficulty, a small
branch of the anterior crural nerve, which
crossed it in front being cut away, and
the ligature, which was single, " passed
through the eye1 of a small needle, and
brought through the integuments an inch
distant from the incision, on the outside."f
In the evening the bandage was a little !
stained with blood, but he went on very ]
well until the 8th, when the house-surgeon ^
perceived a slight pulsation in the tumour. 1
This appeared for several days, more per-
ceptible at one time than another, but
never very distinct, and finally appears
to have ceased. On the 17th, the liga-
ture could be drawn out two inches, and
on the 18th, it was found loose among
the dressings. During the night, secon-
dary hajmorrhage took place, and before
the house-surgeon arrived, eight ounces
of florid arterial blood were lost. The
tourniquet was applied, and when Mr.
Bell arrived, he prepared to tie the vessel,
but on loosening the tourniquet no bleed-
ing took place. He waited an hour more,
and then left directions that compression
should be employed if the haemorrhage
returned. Next day, the wound was
cleaned, and a small clot found at its
bottom, which was not interfered with,
but the limb was rolled from the heel to
the groin, compresses applied, and the
part directed to be kept wet. On the21st,
every thing appeared favourable, when a
flow of ' dark coloured blood' again took
place from the wound, a roller was applied
round the the thigh below the wound,
which was enlarged upwards for about
three quarters of an inch, the artery dis--
sectedfor, and pulled out with a blunt
hook: a ligature was passed around it, but
not tied; the tourniquet loosened, but not
a drop of blood appeared. After waiting
some time, without any haemorrhage, the
ligature was secured, the wound dressed,
and the limb rolled. On the 3d Decem-
ber, 14 days after the application of the
ligature, it separated, the wound dis-
charging a good deal. From this to the
15th January, he went on pretty well,
the wound, however, being extremely
indolent, and suppurating rather freely,
but on the latter day, a haemorrhage of
what appeared to be venous blood took
place. It soon ceased, and since that
period has not returned, although the
wound still remains fistulous.
The reader cannot fail to be struck by
the similarity between this case, and the
recent one of Mr. Brodie's, about which
so much has been said. It is very difficult,
perhaps impossible, to assign any satis-
factory reason for the haemorrhage in ei-
ther case, but no doubt if the ligature had,
by some chance or other, been applied in
this instance, within an inch or so of the
profunda, certain impartial gentlemen
would have " ejected" their tive or six
pages of abuse on the occasion. Mr. Bell,
* Med. Gazette, No. 13. t
?f- The object of this, we imagine, is? ]
not to disturb the adhesion ot' the wound 1
by the ligature lying in it.?Ed. 1
1828]
Popliteal Aneurism. 565
in a clinical lecture, delivered partly on
this and partly on some other cases, has
touched upon the question of haemorrhage
generally, and we shall take the liberty
of noticing a few of his remarks.
Mr. Bell commences his lecture by
some observations on the value of clinical
instruction, in which we entirely agree,
and only wish that it was more general
than it appears to be. Mr. 13. then
alludes to the circumstances, both of them
connected with death from uncontrolled
haemorrhage, which first drew the atten-
tion of the celebrated Guattani, and that
modern " Tamerlane of surgery," as
Dr. Barry very appropriately terms him,
Sir Astley Cooper, to the study of their
profession. These are pleasant topics,
but alas 1 the dulce is not for critics, and
we must pass them over, as we must a
well told hccruorrhagic adventure of Mr.
Charles Bell's own, occurring at?
thai dread hour,
When churchwards yawn, and graves send forth
their dead.
Mr. Bell asks himself, "how is a bleed-
ing artery stopped ?" and then proceeds
to answer in the following manner. The
blood is retained fluid " solely through
the influence of the living vessel which
contains it." This nescio quid, or living
influence, according to Mr. B. resides in
the inner coat, " for the moment the
blood escapes from it, coagulation com-
mences." This position is dwelt on
again and again, and is illustrated by
the well-known fact, that if an artery be
merely cut or punctured, it will continue
to pour out its blood ad deliquium, but
if violently torn or contused, will fre-
quently not bleed a drop. In the first
instance, that of the clean cut, " the
artery is opened without the disturbance
of that principle of life, which preserves
the blood liquid, and therefore the blood
continues to flow," whilst in the latter
case, we suppose the principle of life is
disturbed, and therefore the blood does
ilot continue to flow. In short, Mr. Bell
conceives that the most important agent i
in the arresting of haemorrhage, " is the
influence of life which resides in the 1
coats of the vessels and in the blood," '
and he laments, that this " essential" :
principle has been overlooked, and neg- 1
lected by his contemporaries. j
Now we are not among those who <
seem to consider the human bodv as a <
mere druggist's shop, a decoction going
on in this organ, and a fermentation m
that, but we do think that it is somewhat
late in the day to revive the doctrine of
the life in the blood. Upon the consider-
ation of this theory we shall not enter,
because, we believe, the opinions of me-
dical men are pretty well decided on the
subject; but we will just examine the
power of this influence of life which re-
sides in the inner coat of the blood-ves-
sels. There cannot be a doubt that, in
the greater number of aneurisms, the
inner coat of the artery is ruptured, and
this is a support, as far as it goes, to Mr.
Bell's position. In many cases, however,
the inner coat most certainly is not rup-
tured, and yet coagulation has taken place
within the dilated vessel. Again, suppose
the carotid artery dilated at its origin,
and suppose a surgeon is called in, who
resolves to immortalise himself by the ope-
ration of tying the vessel ultra tumorem.
Well; the ligature is applied, the sur-
geon immortalised accordingly, and the
patient dies, at the expiration of a certain
space of time, of secondary haemorrhage,
from the upper portion of the artery.
Upon dissection, the dilated portion of
the vessel is found filled with coagulum,
although the coats, inner and outer, are
perfectly entire. We will take another
instance ; Mr. Bell, not liking the look
of a ligature ultra tumorem, performs the
operation for aneurism in the common
way, or ties the artery on the face of a
stump. This patient also dies, and the
artery tied is found filled with coagulum,
from the spot at which the ligature was
applied, up to the origin of some large
branch, a distance, we will say, of an
inch and a half. These are not hypo-
thetical cases, but in all of them is found
a coagulum, and often a very consider-
able one too, where the inner coat of the
artery was perfectly entire. Perhaps it
maybe said, " oh! but when the ligature
is applied, an injury is done to the ves-
sel, and the principle of life in its inner
coat gets a shock, which disturbs the
influence it naturally exercises on the
blood." If this were the case, we should
be glad to know why coagulation should
stop at the origin of a large arterial
branch, as when the femoral artery is tied
immediately below the giving off of the
epigastric, in which case there is little
or 110 coagulum at all. But we meet
566 Periscope; oh, Circumspective Review. [March 22
with a still more unexceptionable instance
of coagulation taking place within an
artery whose inner coat is sound, namely,
in what is called the spontaneous cure of
aneurism. In this case, the coagulum
forms not only in the aneurismal sac, but
in the vessel leading to and from it,
frequently to a considerable distance.
From these facts it is evident that this
living influence residing in the inner coat
of the blood-vessels is purely imaginary,
and that whenever the blood is placed out
of the current of the circulation, be it in
an aneurismal sac, a dilated artery, or
merely a tied vessel, it coagulates, or, if
you will, it dies. We are willing to
allow that coagulation takes place more
speedily and effectually out of a vessel
than in it; but this is a very different
thing indeed from that mysterious agency
of avis vitse in the blood or blood-vessel,
which it is so easy to talk about, and so
hard to understand.
We have dwelt so fully on this point,
that our limits will scarcely allow us to
allude to the other preventives of haemor-
rhage, namely, the retraction of the ar-
tery?the contraction of its orifice?and
the formation of a coagulum within it.
With regard to the first, it is exceedingly
important, as every body knows, in its
effects, and when prevented, as in the
nutrient canal of a bone, &c. often oc-
casions serious consequences. Mr. B. ob-
serves that he has seen, in compound
fracture of the leg, the nutritia tibiae di-
vided, and bleeding so profusely, as to
induce the consultants to believe that
the posterior tibial artery was wounded.
The contraction of the mouth of the ves-
sel Mr. Bell makes to depend exclusively
on the action of the muscular fibres, but
we doubt whether the elasticity of the
coats does not also come into play. Upon
the coagulum, which forms at the mouth
of a divided vessel, stretching within it
and around it, and forming a kind of cap
upon its extremity, Mr. Bell offers no-
thing new. He recommends the surgeon
not to trust to it in amputation of the
breast, &c. but carefully to tie each vessel,
and encourage the bleeding at the time,
by warm sponges and the administration
of a little wine, so as not to expose the
patient to all the alarm and suffering at-
tendant on secondary haemorrhage. In
this sentiment, we believe, most surgeons
of the present day are agreed.
4. Chloride of Lime.
Mr. Davidson, of Glasgow, has published
a few observations on this corrector of
putrid effluvia in the first number of Mr.
Mackenzie's Journal.
The following extract contains the
particulars of this communication.
" It is an opinion entertained by many
medical men, that the fcetor of intestinal
gases depends upon the presence of sul-
phuretted hydrogen. Now, though it had
not been proven by analysis, that sulphu-
retted hydrogen is seldom found a con-
stituent of these gases, yet analogy would
lead us to conclude that their fcetor de-
pended solely upon the presence of putrid
effluvia. For, if a portion of excremen-
titious master be mixed with a solution
of chloride of lime, the foetor is instantly
destroyed, in the same way as it would
be, if the solution were applied to a
sphacelating ulcer, or mixed with putrid
oil. That putrid effluvia are totally dif-
ferent from contagious miasmata, is very
evident; because the first can, with great
facility, be detected by our senses, and
exist in bodies, which have no infectious
properties ; whereas the existence of the
latter can only be inferred from induc-
tive reasoning. And it is a begging of
the question to maintain, that because
chloride of lime destroys putrid effluvia,
it must therefore necessarily destroy con-
tagious effluvia, because both exist at the
same time in the same animal body. It
may be true that the chloride of lime can
destroy contagious effluvia; but till it be
demonstrated by rigid experiments and
observations, we have no right to draw
conclusions from such vague and unphilo-
sophical analogies.
" There is no substance which destroys
putrid effluvia so thoroughly as chlorine,
or its compounds. Chloride of lime was
first used in France, for this purpose,
which certainly forms not one of the least
important applications of this extraordi-
nary compound.
" The following account is an abstract
of a few cases in which the chloride of
lime has been used:?J. S. has been af-
fected with cough and expectoration for
about six years, and is considerably ema-
ciated. On the 25th October, 1826, he
was affected with sloughy ulcerations in
his gums, which emitted a very fetid
1828] Fever. 567
odour. A gargle was prescribed, in the
proportion of two grains of chloride of
lime to an ounce of water. This, used
occasionally, kept the fetor in check, and
the ulcerations cicatrized in about eight
days. 2d. Mrs. C. in November, 1826,
used mercury for a hepatic and dropsical
affection, to the extent of copious sali-
vation. A gargle, in the proportion of
two grains of chloride of lime to an ounce
of water, was used ; which produced con-
siderable smarting. It was afterwards
reduced in strength, and a portion of
refined sugar was added, which removed
in a great measure its harsh taste. It was
* continued for a fortnight, and when used
frequently, always had the effect of mo-
derating the fetor. The same treatment
has been adopted uniformly in all other
similar cases, and with similar effects.
3d. Mr. M. was affected in December,
1826, with diarrhoea; faeces dark and very
fetid. One teaspoonful of chloride of lime
was ordered to be dissolved in about six-
teen ounces of cold water ; and the feces
to be discharged into the solution. He
used it in this way for ten days, and
affirmed, that in all cases it had the de^
sired effect of destroying the fetor. This
plan has been adopted in a variety of
cases, during the late dysenteric epidemic,
and in several cases of fever. 4th. A boy
about twelve years of age has expecto-
rated about four ounces of pus every day
for three or four years. It seems to be
discharged from a cavity in the right side
of the chest; for the sound emitted on
percussion is very dull in that situation.
He expectorates the whole of the pus in
a few minutes, and it occasions a very
disagreeable fetor in the apartment. But
by discharging it into a strong solution of
chloride of lime, and afterwards gargling
his mouth with a similar solution, much
of the disagreeable fetor is destroyed.
5th. A blacksmith in Nov. 1826, received
an injury on one of the joints of a finger,
which produced extensive and fetid sup-
puration of the joint. The fetor was
completely destroyed by moistening the
dressings, (before they were removed,)
with a solution of chloride of lime, in the
proportion of two grains of the salt to
one ounce of water. 6th. Mrs. V. was
delivered in February last of her first
child. The labour was very tedious, and
there was high excitement during the
latter stages. On the third day after de-
Hvery, she was affected with fixed pain
in the uterine region, and very fetid lo-
chial discharge. A solution of about two
grains of chloride of lime to an ounce of
water, was ordered to be injected into
the vagina, two or three times a day.
This treatment removed the disagreeable
odour 5 and she completely recovered in
about ten days. 7th. Mr. M. whose breath
is naturally fetid, was directed to gargle
his mouth and brush his teeth with a so-
lution of chloride of lime, in the propor-
tion of four grains of the salt to an ounce
of water, with the addition of a small
portion of refined sugar. It had the effect
of removing the fetor of the sordes which
accumulated about his teeth. 8th. J.L.
died of diseased liver and dropsical effu-
sion into the abdomen. On laying open
the abdominal cavity, a very fetid odour
made its escape ; and adhered very tena-
ciously to the hands. After the dissection
was finished, they were thoroughly mois-
tened with a solution of chloride of lime,
in the proportion of about six grains of
the salt to an ounce of water, and after-
wards washed with soap and water. To
medical men, this is an important appli-
cation ; for after dissections, the fetor
often adheres to the hands, most tenaci-
ously, for many hours. A solution of a
similar strength was poured upon the
body, and the clothes in which it was
wrapped were also moistened."
Dr. D. strongly recommends the use of
this substance, both in public and private
practice.
" In ptyalism," says he, " it is fre-
quently of great consequence to many a
young man, to remove the fetor of his
breath, and to prevent the consequent
discovery of his disease ; and even where
there is no reason for concealing the use
of mercury, how agreeable to the feelings
of the attendants would be the neutra-
lization of such offensive putridity I"
5. Fever.
Three evenings of the Westminster Me-
dical Society have been taken up in the
discussion of the nature, seat, and treat-
ment of fever. The subject was brought
forward by Dr. Barry, on the basis ot
Dr. Clanny's experiments on the blood
of typhous patients, alluded to in the 4th
568 Periscope ; or, Circumspective Review. [March
Fasciculus of this Journal, and the humo-
ral pathology was ably maintained by the
former gentleman. We are quite unable
to enter into the details of the arguments
brought forward by the Humoralists and
Solidists, on this occasion. All were
agreed on one fact?the vitiation of the
fluids in the course of the fever ; but the
grand point of contention turned on the
question of priority, as to the nervous or
vascular systems being the primary seat
of fever?or, in other words, did the re-
mote cause, say contagion or malaria,
make its first impression on the nerves
or on the blood-vessels ? Dr. Barry, of
course, argued for the latter; while Dr.
Copland and Dr. Thomson contended for
the neurological pathology of fever. In
the second night's debate, Dr. Copland
took an opportunity of fully developing
his doctrine of fever, which supposes the
morbific cause to make its first impres-
sion on the system of ganglionic nerves
?especially on those distributed to the
air passages in the lungs. From this
source, he conceives, the morbid impres-
sion is transmitted to the cerebro-spinal
system of nerves, and consequently that
all the functions dependent on both sys-
tems become ultimately deranged?and
the fluids contaminated. Dr. Thomson
maintained, that the first impression was
made on the nervous system?and that
all the derangements of function and
vitiation of fluids which followed, were
effects of this morbid state of the nerves.
Dr. Johnson argued, that an accurate ob-
servation of the phenomena which pre-
sented themselves to the senses, after
the application of the cause of fever,
whether a contagion, a specific poison,
as that of small-pox, plague, &c. or a
malaria, would not enable any unbiassed
man to determine, that the nervous sys-
tem was more particularly or more early
implicated than the vascular apparatus.
It was true, the phenomena of sensation
came more clearly under our view at the
beginning of fever, than any changes in
the fluids could possibly do. We could
hear a man complain of lassitude or pain
in his limbs, but we could not see any
poison that might have been floating in
his veins, and acting on the nervous sys-
tem for days previously. The period of
incubation, or that period of apparent
health which intervened between the ap-
plication of a poison, say small-pox con-
tagion, and its open manifestation, indi-
cated that some contamination of the
fluids was going on secretly all that time.
Mr. North, Dr. Ley, and Mr. Lambert
declared their opinions, that the subject
of discussion was totally useless, and
could never bear, in the remotest degree,
on our practice at the bed-side. This
damper on the " march of intellect" was
somewhat indignantly met by Dr. Barry
and Dr. Thomson, who, though oppo-
nents in the controversy, now joined hear-
tily in defending the propriety?the uti-
lity of discussions on the nature, seats,
and causation of diseases, even although
such discussions might not lead to a com-
plete elucidation of any of the points
discussed. In this sentiment the majo-
rity of the Society seemed to agree.
In the course of this discussion some
therapeutical observations came out,
which are not undeserving of attention.
Dr. Copland advocated the propriety of
emetics in the incipient stage of fever,
as a remedy by which he had, in nume-
rous instances, arrested at once the pro-
gress of the disease. On the other hand,
he had seen oil of turpentine save the
lives of great numbers, in the last stage
of exhaustion, when no other remedy
offered the slightest chance of success.
Dr. Barry adverted to the very opposite
plans pursued by the Portuguese phy-
sicians and the disciples of Broussais. In
the Portuguese hospitals,there is an emetic
room, into which the fever-patient walks,
as a matter of course, when he first arrives,
and there undergoes the necessary disci-
pline. After the emetic, he has chicken-
broth, and saline draughts alternately,
through the whole course of the disease.
Bleeding or any strong medicine is rarely
thought of?and the general result is as
favourable as under the most vigorous,
depletive measures of British practiti-
oners. On the other hand, he has fol-
lowed M. Broussais for weeks and months
?he has seen his patients starved or
leeched to death, so that, on dissection,
any man might have read the smallest
print through the coats of their intes-
tines ! Broussais, of course, abhors the
Portuguese emetic plan. Indeed he gene-
rally promises to cure all those who have
not been poisoned by emetics and pur-
gatives. Seeing, then, such diversities
of opinion, and such empirical modes of
treatment pursued, was it not (he said)
1828] . Erysipelas. 569
desirable to calmly investigate the seats
and causes of fever, in order that the
minds of youth and of practitioners might
be emancipated from the fetters of em-
piricism at least, if they could not arrive
at the exact truth?
In the third night's discussion, the de-
bate was principally carried on between
Drs. Ley, Copland, and Barry. Thera-
peutics formed the prominent subject of
investigation, and the efficacy of oil of
turpentine in puerperal fever was warmly
advocated by Dr. Copland, who seems to
have had great success with this remedy
In the latter stages of what he called
malignant puerperal fever. He seldom
gave more than half an ounce or an
ounce in a draught?or double that by
enema?and, in general, he did not give
it more than twice in the same case.
Fomentations of the same were also used.
Dr. Ley seemed to view the remedy as
one which relieved a particular and very
distressing symptom of the disease?tym-
panitic distention of the abdomen. For
such it was an admirable remedy; but
he did not appear to consider the oil of
turpentine as possessed of any specific
powers against the dangerous disease in
question. The utility of theory in fever
was again agitated in the Society, and
Dr. Barry, Dr. Copland, Dr. Ley, Dr.
Johnson, and others, took active parts in
the discussion.
6. Literary Justice.
Our readers will remember the charge
made by Mr. Lawrence against Dr. John-
son, in our 4th Fasciculus, and Dr. John-
son's answer to that charge. The Lancet,
-with its usual j ustice, publishes the charge,
?but suppresses the reply! In place of
the latter, the Lancet favours its readers
with ten columns of blackguard abuse
of Dr. Johnson, including, at least, one
whole column of downright falsehoods !
If we gave the Lancet a large weekly fee
for enhancing the character of this Jour-
nal and damning its own, no surer me-
thod of effecting both objects could be
pursued than that which it has adopted.
We earnestly entreat each and every of
our readers to peruse the twelve columns
(it always dedicates twelve columns
to us) between page 448 and page 454,
Voc. VIII. No. 16. 3 P
of the Lancet, No. 23(5, as the greatest
compliment that this Journal has ever
received since its first commencement.
In what a predicament has the Lancet
placed Mr. Lawrence ! It publishes his
statement?suppresses ours?and substi-
tutes ten columns of abuse, lies, and
garbled extracts ! " There is such a
thing as common sense ;" and although
there may be no justice in the Lancet,
there is justice in the minds of profes-
sional men. For this last act we return
unfeigned thanks to the Lancet. That
journal has done Dr. Johnson more real
service than all his friends put together.
Perge pede quo cepisti.
7. Erysipelas.
Sir A. Carlisle has published some sen-
sible remarks on this disease in the 14th
number of the Medical Gazette, not,
however, unmixed with some fanciful
opinions. We find him an advocate for
the union of physic and surgery?(a
union which we have long advocated)?
for the constitutional origin of erysipelas
?for the humoral pathology of the disease
(as " arising from vitiated blood")?and
for its occasional epidemic character.
" Assuredly (says he) the common source
of erysipelas is improper diet, through
which the blood is supplied with crude
or noxious materials." Sir A. is fully
convinced that excitement in the system,
as from catching cold, only " gives ac-
tivity to a previously existing morbific
humour."
" Those violent eruptions, vulgarly,
but not illogically, called surfeits, are
the immediate consequences of one per-
nicious meal; and they generally display
Erysipelas in its most malignant charac-
ter j while moderate and habitual errors
in diet mitigate the acuteness of the dis-
ease, and keep the patient in a state of
continued liability to relapses, whenever
exposed to any extraordinary vicissitude,
and which thence assume either a mild
or chronic type."
. It must be allowed (as Sir A. justly
remarks) that erysipelas is frequently
first observed as a local affection, and
apparently limited to the corion or true
skin; "but the eruption is always pre-
ceded by some derangemcut in the ali-
570 Periscope; ok, Circumspective Review. [March22
mentary functions, and, for the most part,
by loss of appetite, nausea, and consti-
pation, followed by the other signs of
incipient fever." As far as idiopathic
erysipelas is concerned, we believe Sir
A's observations are correct; but there
are cases of traumatic erysipelas, where
we cannot always trace this preceding
disorder of the digestive apparatus. A
man, not perhaps in sound health, or of
an irritable constitution, receives a wound
or hurt, and a few days afterwards, ery-
sipelatous inflammation spreads from the
injured part. " Perhaps all the varieties
.of erysipelas, says the author, are to be
ascribed to the several deviations in the
acrimony, or in the quantity of the ex-
uding morbid humour, and in some de-
gree, to the constitutional habit of the
patient." Sir A. having examined the
serous effusion under the skin, in a great
number of cases of erysipelas, " always
found it to contain a free acid." The
following, in a few words, is Sir A's pa-
thology of the disease in question.
" My views of Erysipelas, thus ma-
tured, are, that it is a humoral and con-
stitutional inflammatory disease, occasi-
oned by alimentary crudities; and because
certain vegetable acids, and acidifiable
viands, are often the notorious antece-
dents of the disease. I believe, as before
stated, that the dominant error in the
morbid fluids is acid."
We shall subjoin our author's thera-
peia. He does not like venesection, and
prefers cupping, in plethoric habits; but
the following is the plan of treatment on
which he seems to depend.
" When the attack has been sudden,
and the stomach is known to be loaded
with crude food, an emetic of ipecacuanha
is adviseable, and it should be immedi-
ately followed by a cleansing cathartic,
given in reiterated doses until the lower
bowels are thoroughly emptied. If the
patient be of a full habit, and the pulse
ample, abstinence from food and drink
should be recommended, because the func-
tions of the stomach are especially dete-
riorated in Erysipelas, and diluents pro-
mote all the alimentary fermentations.
Mercurial purgatives are objectionable,
because they produce putrescent excre-
tions ; and also, because of the uncer-
tainty as to their injurious constitutional
effects on certain individuals. Saline
cathartics seldom cleanse the bowels ef-
fectually, and they mischievously aug-
ment the fluidity of the aliment.
" The purgative which I ordinarily ad-
vise is composed of one drachm of pow-
dered jalap, two scruples of sulphate of
potash, and half a drachm of carbonate
of soda, mixed with eight ounces of in-
fusion of senna; of this two or three
table-spoonsful should be taken every
second or third hour, until copious and
cleansing evacuations are procured. From
a conviction that alimentary acidity is
generally prevalent and requires to be
subdued, I give from ten to sixteen grains
of sub-carbonate of soda every four hours,
in barley water. If the mouth is parched,
soda water proves very refreshing. Where
great debility is obvious, the carbonate
of ammonia, in doses from five to eight
grains, should be preferred to soda; and
three or four grains of aromatic powder
may be joined with each dose."
As might be expected from the doc-
trine maintained by Sir Anthony, the
local treatment of erysipelas is a very
subordinate consideration. Free incisions
are recommended, where the subcutane-
ous tissue has been " invaded by the
morbid secretion." When sphacelus has
taken place, the chloride of lime is spoken
of in terms of the highest applause.
" This estimable compound arrests the
putrefactive fermentation ; by which not
only the local condition of the mortified
parts, but the general health and com-
fort of the patient, are essentially bene-
fitted." Dietetic regimen is strongly
insisted on by Sir Anthony. Although
much inclined to the humoral pathology,
we can hardly help suspecting that the
acid theory of the author is more fanciful
than sound We agree with him in the
general principles of treatment, except-
ing his futile objections to mercurial pur-
gatives ; and also in the view which he
takes of the constitutional origin of the
disease.
8. Mr. Abernethy's Lectures-
These lectures are now published through
two, if not more channels?and they are
still delivered, annually, to the largest
class of students that is to be seen in this
metropolis. That the doctrines, opinions,
and practices promulgated and inculcated
through these lectures, must have a very
1828] Mr. Abernetiiy's Lectures. 571
extensive influence on the profession, es-
pecially on the junior orders, cannot be
questioned ; and if the matter of the lec-
tures presents legitimate subjects of dis-
cussion, or even criticism, we think that
no publication can better deserve an ex-
amination than that which is most uni-
versally read and most generally influen-
tial. We shall, therefore, take up the
various diseases treated of in these lec-
tures, promising that our comments shall
be guided by no other feeling than a de-
sire to establish truth.
Physic and Surgery?Fever.
Mr. A. upholds the " unity and indivi-
sibility" of physic and surgery?and then
goes on to maintain, that the symptoma-
tic fevers arising from local injuries are
" exactly like those which Cullen has des-
cribed under the heads of synocha, syno-
chus, and typhus?and no physician can
distinguish them from those which occur
without any external injury." Again, he
says?" I will venture to assert, that the
fevers produced by local disease, are the
very identical fevers which physicians
meet with where there is no external in-
jury." This doctrine we hold to be equally
erroneous and dangerous. It presumes
that the causes of fever make no difference
in the character of the disease. This is
a great error. Look at two men, one of
whom receives a bayonet-wound in the
chest?the other a malaria in Walcheren
??or the contagion of typhus in the pur-
lieus of Saffron Hill. Will the period of
incubation, the invasion, the nervous and
vascular phenomena, the duration, or
the termination of the two fevers be iden-
tical ? No ! Is the fever resulting from
the contagion of typhus, or the miasm of
the fen, as much within the control of
art, as the symptomatic fever arising from
the wound in the lungs ? Can we " put
out" the typhus fever in two, three, or
four days, by venesection, not measured
by ounces, but by wash-hand-basins full ?
No, verily. If, then, the character, the
course, and the treatment of the idiopa-
thic and the traumatic fevers, are dift'er-
ent, as we maintain them to be, how is
Mr. Abernethy justified in the sweeping
assertion, that they are identical ? It is
very true that, towards the fatal close of
all fevers, whether symptomatic or idio-
pathic, the features approximate, and the
character of the morbid phenomena be-
comes nearly, if not wholly, identical.
But this is no fair reason for confounding
them all together. The very description
which Mr. A. gives, at page 7 of his first
lecture, negatives its application to the
great class of malarious and contagious
fevers. There is no similitude between
the symptoms of these last, and those re-
sulting from a compound fracture, as de-
lineated by our author.
Mr. Abernethy next proceeds to what
is called the " ratio symptomatum" of
sympathetic fever, which is summarily
dispatched in the following manner,
" Hang it, I need not trouble you with
this ; the symptoms explain themselves ;
they are the natural consequences of the
excitement of the heart and arteries, and
the disturbed state of the digestive organs."
This is certainly a very easy way of cut-
ting the Gordian knot! The whole ra-
tio symptomatum in fever is explained by
excitement of the heart and arteries?and
disturbance of the digestive organs. Whe-
ther this disturbance is a cause, or an
effect, of the local and constitutional af-
fection, we leave our readers to judge.
It would not be worth our while to enter
into an examination of this point, in the
present instance. The treatment is still
more easily managed.
"Treatment! there is no treatment.
The disease is the necessary and natural
consequence of the injury : it must in-
evitably take place :?You may mitigate,
but you cannot cure it." What! there
is no treatment?yet "you may mitigate"
the disease ! Is this not the treatment in
the great majority of diseases? Can we
in any case, take the ratio medendi out
of the hands of Nature, and cure the dis-
ease by the main force of art ? The lec-
turer goes on to say, that we must bleed
in symptomatic fever; but really his ob-
servations are calculated to destroy the
manufacture of lancets, to starve the in-
creasing population of cuppers, and to
revive the trade of leech-catching !
" Do not take away his blood, which
is his life, for you may find after a certain
time, that he will stand in need of every
degree of vital energy to recover from the
injury. I have seen a patient bled, and
bled, and two or three days after, the me-
dical man has been glad to throw in the
bark, and try every means, when it is top
572 Periscope; or, Circumspective Review. [March 22
late, to give strength and vigour to the
constitution of the patient."
Again, Mr. Abernethy says :?
"As the fever subsides spontaneously
on the third or fourth day, when suppu-
ration takes place, avoid bleeding the
patient if possible. Give him diluent
drinks and acids, such as lemonade, &c.
for which he has a desire."*
Cleansing the bowels is properly in-
sisted on, as a necessary measure. But,
as the lecturer grounds his observations
on a case of compound fracture, and
that in a London subject, where he does
not think a healthy subject is to be
?found, it is manifest that the didactic
precepts, as far as regards fever, are
extremely imperfect?not to say erro-
neous. London is not spread over the
whole surface of the earth?and injuries
are not confined to compound fractures
of the legs of drunken draymen in the
vicinity of Bartholomew's Hospital.
What would have become of our soldiers
and seamen in the late war, if the fever
induced by the wounds of bayonets, bul-
lets, and splinters, had been treated
according to the economical principles
inculcated in these lectures ? If our
medical officers had acted on these narrow
principles of economy, in respect to blood-
letting, we can only say that they would
have verified the old adage?" penny wise
and pound foolish." The lecturer re-
marks that purgation, by promoting the
secretions, lessens the plenitude of the
blood-vessels. " This is a fact which
those who have not studied anatomy and
physiology cannot be so thoroughly ac-
quainted with as those who have." The
ancients were well acquainted with this
fact, although very imperfectly versed in
anatomy and physiology. But leaving
this on one side, who are those against
whom the above sarcasm is levelled?
Against physicians, of course ! They
can be supposed to know nothing of
anatomy and physiology. Yet if we go
into the medical societies of this or any
other metropolis, we shall find that phy-
sicians are not behind the level of their
surgical brethren, in anatomy, physiology,
or pathology. No class of the profession
should be singled out for animadversion.
He who cultivates his art should be reve-
rencedj be his designation what it may?
he who does not keep himself on a level
with the progress of science should be
stigmatized, even if he were a teacher of
the art. Medical knowledge is not res-
tricted to names, grades, or ranks. It is
a republic in which title will not
always screen ignorance, nor humility
always prevent the rise of merit.
9. Influence of the Stomach on
the Brain.
In a former paper we gave some account
of a dissertation by M. Bayle, on the
influence of chronic inflammation of the
stomach and bowels in the production of
mental alienation. In a second memoir,
he adduces further examples of this in-
fluence, and winds up with certain con-
clusions or reflexions, which we shall
briefly notice.
* Is it not astonishing that a man, pro-
fessing to be an accurate obserrer of na-
ture, and a critic on the opinions of the
most eminent physicians of the world,
should maintain that a fever " which
subsides spontaneously on the third or
fourth day," is identical with that which
physicians meet every day where there is
no external injury?in other words, that
this fever from a wound is the same as
that from contagion or malaria ! ! Is
this a doctrine to guide the rising gene-
ration of pupils, when they go into the
country to practise their profession ?
It is true that Mr. A. apparently recol-
lecting himself, says?" if a man be shot
thi/ough the body?if an internal and
vital organ is injured and inflammation
come on that organ," you must bleed
repeatedly and decisively. " We do not,
however, bleed here for fever, but for
inflammation." Now by this passage,
Mr. A. virtually gives up the identity of
idiopathic and symptomatic fevers. Ac-
cording to this doctrine, we have no
warranty for venesection, however high
the symptoms of fever may run, unless
we have evidence of inflammation of a
vital organ ! We are not to bleed for
fever?but for inflammation. Mr A.
ought to know that most violent fevers
commence without inflammation?and if
we did not bleed, in such cases, inflam-
mation would assuredly supervene in
gome organ or structure. The doctrine
is decidedly bad.
1828] Asthma?Irritability of the Lungs. 573
lino. In a few cases, chronic phlegT
masia of the mucous membrane of the
stomach and bowels, is capable of in-
ducing mental alienation?and, in many
cases, it is capable of keeping up the
mental malady, and modifying its cha-
racter.
2ndo. Most of those who become de-
ranged at the close of chronic gastro-
enteritis, have inherited a constitutional
predisposition to the disease from their
parents. Their mental faculties are gene-
rally weak?the brain irritable. The dys-
peptic and nervous sufferings of people
labouring under gastric and intestinal
irritation, tend very much to mental
hallucination.
3tio. The anatomical characters of the
gastro-enteritis which accompanies or
causes mental alienation, does not differ
from that which is unaccompanied by any
mental aberration.
4to. The manner in which the gastro-
enteric affection acts on the head is two-
fold. When the pain of the abdominal
malady is great, it is sympathetically
propagated to the brain, and there dis-
turbs its functions, in the same way as it
is propagated to the heart, and produces
the phenomena of fever. Hence the
symptoms of mania. But when the
gastro-enteritis is chronic, and less in-
tense, or the cerebral system of the
patient less disposed to irritation, then
the dyspeptic sufferings of the individual
gradually dispose to hypochondriacism?
and hence arise various kinds of mono-
mania?and especially the dread of being
poisoned, with obstinate refusal of food,
&c. These two forms differ only in
degree, and they often pass from one
into the other.
5to. This dread of poison and obstinate
refusal of food are regarded by M. Bayle
as the most constant and essential symp-
toms of mental alienation accompanied
by chronic inflammation of the mucous
membrane of the digestive tube. The
two symptoms above-mentioned are " the
expressions of gastric and intestinal suf-
ferings in mental alienation." They may
exist, he observes, without this condition
of the stomach or bowels, but he has never
seen a case where they were present,
and where the symptoms of chronic gas-
tro-enteritis did not shew themselves
during iife, or the traces of it were found
after death.
6'to. It follows from these premises, if
they are correct, that the treatment of
insanity, where these phenomena obtain,
must be specially directed to the gastro-
intestinal affection?and does not differ
from that which is necessary or proper
where no cerebral disturbance is com-
plicated with the original malady. This
treatment hinges almost entirely on re-
peated leecliings of the epigastrium?
mucilaginous drinks?extremely abste-
mious regimen?exercise in the open air
?counter-irritation.?Revue Meuicale.
Fanciful as the above doctrine may
appear to those who look no farther than
the organ apparently most affected, for
the source of a disease, we believe there
is some foundation for it in fact. The
mental miseries resulting from gastro-
intestinal irritation are but very imper-
fectly known even to the most experienced
physicians. Nothing, in short, but actual
personal sufferings can teach the terrible
but instructive lesson !
10. Irritability of the Air-cells?
Asthma.
A perusal of Mr. Abernethy's lectures,
as now published through more than one
channel, is calculated to excite various
emotions. There is a mixture of accurate
observation, eccentric, not to say errone-
ous deduction, shrewd remarks, and hu-
morous naivete, which is altogether un-
paralleled in didactic instruction, and me-
dical literature. It is our intention to
give a running commentary on these lec-
tures, which we hope to render somewhat
interesting. The present subject is irri-
tability of the mucous membrane of the
air-cells, as one of the principal causes
of that mysterious disease asthma.
Of the irritability of this membrane
Mr. A. entertains no more doubt than of
the irritability of the urethra?of which
there can be no doubt.
" Ordinarily, respiration may be said
to be a mechanical process; we enlarge
the capacity of the chest by the in-
tercostal muscles, and the air is forced
into the lungs : we diminish the chest,
and the air is forced out of the lungs, just
as if you were using a pair of bellows.
Ordinarily, respiration is carried 011
merely as a mechanical process j but,
574 PjittiscoPE; or, Circumspective Review. [March 22
extraordinarily, do we not find manifes-
tations that air cannot get into the lungs,
though we do endeavour to enlarge the
chest ? You know you never could lift
up the board of the bellows, if you were
to stop up the holes that admit the air,
and why ? On account of the immense
weight of the atmosphere. Just so, if
you were to put a rope round aman's neck,
and stop the air from entering into the
trachea ; it is not the strongest man that
ever lived that could afterwards enlarge
that man's chest; to do that, would be
to lift up an immense load of air. A
man having irritable lungs, may be sitting
comfortably enough at the fire-side, but
a little smoke comes into the i-oom, and
he can breathe no more : he gasps for
breath, he cannot enlarge the chest, and
he finds the utmost difficulty in respiring ;
but where is the difficulty ? Where is
the sensation of pain and contraction ?
Why, in the lungs themselves ; the hin-
drance is there ; I believe it is all irri-
tability."*
Passing over the error that respiration
is carried on by the intercostal muscles
alone, we cannot but see the want of
analogy between the bellows and the
lungs in this case. If, indeed, Mr. A.
had said that irritation of the mucous
membrane of the glottis, might throw
the surrounding muscles into a state of
spasm or contraction, and thus prevent
the ingress of air into the lungs, we could
readily understand him ; because it is in
this way that irritation of the urethra
causes temporary or spasmodic stricture
of that canal. But, when he says that
irritation of the mucous membrane lining
the air-cells causes contraction of the
said cells, and shuts out the air, we are
at a loss for the quo modo?unless he can
demonstrate muscular fibres in the pa-
rietes of these cells. We are inclined to
think that the true explanation will be
found in irritation of the membrane lining
the passages leading to the lungs (and
which, as we said, are surrounded by
muscles) rather than in the air-cells
themselves. Be this as it may, it is very
difficult to account for the phenomena
presented by what are termed asthmatic
people. Pure air generally relieves the
dyspnoea, where there is no organic dis-
ease?but not always. Asthmatic people
of London go about the streets " with
their shoulders hitched up to their ears,
and using every auxiliary to enlarge their
chests." Mr. A. instances the case of a
neighbour whom it was painful to see
walk about, while breathing an impure
air, as he appeared to be in imminent
danger of suffocation. Such was his state
in Bedford-row, but by the time he had
got to the top of Gray's Inn-lane, in his
way to his country-house, for a mouthful
of fresh air," he breathed perfectly well."
But there are many others who breathe
better in an impure than in a pure air.
Mr. A. knew a man, " whose lungs were
so asthmatical that he could never lay
down in his bed at night," and he was
advised to go to the South of France.
He consulted Mr. A. " I told him what
I tell every body else, that the best thing
he could do was to take care of the state of
his stomach." In about three weeks, he
called again on Mr. Abernethy, and re-
ported that he had done according to his
directions, and that he had breathed the air
of London for the above-mentioned period
of time, without the least difficulty of
breathing. He went back to his native
air, which was on the top of a high hill
?and he was nearly suffocated the very
first night he slept there. Another man
lived in a room which was filled with
sulphuric acid gas, and found that " it
relieved his difficulty of breathing in an
amazing degree."
" I say that is whimsical; but all this
leads to convince me, that there is a state
of irritability in the lungs, and which
proceeds from the state of the stomach
too."
It is almost needless to say, that this
physiology and pathology of the mucous
membrane of the bronchia and air-cells,
is extremely imperfect, and far from what
we would expect from such a man as
Abernethy. When we find a paroxysm
of spasmodic asthma, as it is called,
lasting for one, two, or three days, and
ending in a copious expectoration, we
cannot but conclude that a turgescent
condition?a congestion, if you will?of
the vessels distributed over this extensive
membrane, had previously existed?and
that Nature had relieved the dyspnoea, by
the discharge of a copious effusion or
exudation from the said membrane, in
the shape of a muco-purulent expec-
* Lectures, page 3/5.
1828] Diagnosis of Hernia. 575
toration. The doctrine of mere irritation
then, is, like many other doctrines, too
confined and exclusive. It is not founded
on an extensive and liberal view of the
phenomena presented by disease.
11. Diagnosis of Hernia.
A very animated discussion on this point
took place at the Westminster Medical
Society, on the evening of the 15th, and
really some observations fell from one or
two gentlemen on the occasion, which,
as the Americans have it, would be ex-
ceedingly important, if true.
The subject was very ably introduced
by Mr. Caesar Hawkins, who considered,
seriatim, the symptoms diagnostic of her-
nia?the tumours which may be mistaken
for it, or combined with it?the contents
of the sac, where a hernia has been proved
to exist?and, lastly, these having been
ascertained, their pathological condition.
From this it is evident, that the field was
a very extensive one, and we are sorry
that our limits will not permit us to do
justice to Mr. Hawkins' observations.
With regard to the symptoms of stran-
gulation, Mr. H. thought that it was ex-
ceedingly difficult to distinguish between
them and inflammation of the intestines,
or ileus, in acute cases; but, in the chro-
nic forms, the diagnosis is by no means
so difficult. Varicocele, or varix of the
veins of the round ligament, a very rare
disease, of which we detailed a case in
our last fasciculus, is not unfrequently
mistaken for omental hernia; but, by
placing the patient in the crect position,
and making pressure at the ring, the des-
cent of a hernia is prevented, whilst the
varices, if they exist, become turgid.
Enlargement of the femoral vein, too,
beneath the crural arch may counterfeit
a femoral hernia. The diagnosis, how-
ever, is similar to that in the former in-
stance, viz. pressure on the groin, which
causes the vein to swell, but prevents the
descent of a herniary tumour. Hydrocele,
encysted hydrocele of the cord, especially
the latter, and an enlarged absorbent
gland, which is situated just within the
external J'ing, may be severally mistaken
for inguinal hernia, whilst it is well known
that surgeons, even of eminence, have
cut down upon a bubo, imagining it to
be a femoral hernia. After noticing va-
rious other sources of error to which the
practitioner is exposed, Mr. Hawkins
proceeded to point out the distinctions
between intestinal and omental hernia,
which he thought of importance, espe-
cially in the more chronic cases. In the
former, when the intestine is closely girt,
purges maybe useless, or worse; whereas,
in epiplocele, they may prove of ser-
vice, and, at any rate, we have more time
afforded us, before having recourse to an
operation. In the acute cases of intes-
tinal hernia, Mr. Hawkins was of opinion
that bleeding and the warm bath should
precede the application of the taxis, as
they tend to diminish the inflammatory
engorgement of the tumour.
Dr. Somerville remarked that he had
frequently seen patients die after the
operation for hernia, performed appa-
rently under favourable circumstances,
when no organic lesion of any con-
sequence could be detected after death.
In such cases, the nervous system ap-
peared to have received a shock, and
the patients to have sunk under that state
which has been designated by the name
of " constitutional irritation." This was
a most unfortunate phrase, for no sooner
was it uttered than Mr. Lambert started
on his legs, and expended we know not
how much valuable argumentation, to
prove that this " constitutional irri-
tation" was a mere non-entity?an ignis
fatuus in the Doctor's brain. Mr. Bennet
observed that there is most certainly
" a something," call it what you will,
which supervenes even on trifling acci-
dents or operations, and carries off the
patient quite independent of any inflam-
matory process. Thus, in opening the
abdomen of dogs, Mr. B. had seen them die
in so short a period, that it was impos-
sible for inflammation to have developed
itself.
Dr. Duffin having related a case in
which he had nearly mistaken an en-
larged and suppurating gland, under very
obscure circumstances, for an inguinal
hernia, was attacked, unguibus et rostro,
by Mr. Lambert, who declared, that the
symptoms of strangulated hernia, were
so decided, that no surgeon who knew
any thing of his profession, ought to
mistake them! Mr. L. was then asked
by Mr. North to detail those symptoms
of strangulation, seeing that they were
576 Periscope ; or, Circumspective Review. [March 22
all so plain and palpable. At this question,
Mr. Lambert seemed rather inclined to
fight shy, but after a little coaxing on the
part of Mr. North, replied that he was
not inclined to place so much reliance on
the local symptoms as the constitutional.
These were obstinate constipation?vo-
miting?small hard pulse?tenderness of
the abdomen. It was observed, however,
that these symptoms might one and all
exist, totally independent of any herniary
"tumour, and depend solely upon intesti-
nal inflammation. In opposition to Mr.
L's comparative neglect of the local
symptoms, it was urged by Mr. North,
with great justice, that surgeons in gene-
ral, are disposed to place very consider-
able reliance upon them, and Mr. Law-
rence, himself, attaches a good deal of im-
portance to the condition of the tumour.
12. Ice to Aneurisms.
? Baron Larrey has lately presented a
?memoir to the Royal Institute, on the
treatment of aortic aneurisms, which con-
sists in the adoption of Valsalva's method
?starvation?and the application of ice
to the region of the aneurism. Among
many instances of success attending this
treatment, we may cite the following.
A soldier, after a wound, became affected
with varicose aneurism of the crural ar-
tery. The volume of the tumour exceeded
that of a man's fist, and "all operation
was impracticable"?foi what reason we
are not told. The patient, however, was
doomed to death, when the method of
Valsalva (starvation, absolute repose, re-
peated bleedings) and the constant ap-
plication were tried. Afterwards moxas
were applied to the tumour. In the course
of a few months, the aneurism was con-
siderably, though very progressively, re-
duced. It was evident that the parietes
of the tumour became gradually thick-
ened. Finally, the canal of the crural
artery was completely obliterated, and a
cure was effected in the course of a year.

				

